# Structural Health Monitoring of Civil Infrastructure Using Optical Fiber Sensing Technology: A Comprehensive Review

**DOI:** 10.1155/2014/652329

**Published:** 2014-07-14

**Authors:** X. W. Ye, Y. H. Su, J. P. Han

**Affiliations:** ^1^Department of Civil Engineering, Zhejiang University, Hangzhou 310058, China; ^2^Key Laboratory of Disaster Prevention and Mitigation in Civil Engineering of Gansu Province, Lanzhou University of Technology, Lanzhou 730050, China

## Abstract

In the last two decades, a significant number of innovative sensing systems based on optical fiber sensors have been exploited in the engineering community due to their inherent distinctive advantages such as small size, light weight, immunity to electromagnetic interference (EMI) and corrosion, and embedding capability. A lot of optical fiber sensor-based monitoring systems have been developed for continuous measurement and real-time assessment of diversified engineering structures such as bridges, buildings, tunnels, pipelines, wind turbines, railway infrastructure, and geotechnical structures. The purpose of this review article is devoted to presenting a summary of the basic principles of various optical fiber sensors, innovation in sensing and computational methodologies, development of novel optical fiber sensors, and the practical application status of the optical fiber sensing technology in structural health monitoring (SHM) of civil infrastructure.

## 1. Introduction

Structural health monitoring (SHM) has been a fast-developing domain in engineering disciplines especially in civil engineering field. The innovation in the SHM technologies as well as the development of the large-scale SHM systems has boomed within the engineering and academic communities over the last two decades [1–7]. The available practical experiences have proved that the progressive advancement of the sensing techniques will undoubtedly expedite the evolution of the SHM technology. In comparison with the traditional mechanical and electrical sensors, the optical fiber sensors possess some unique advantages such as small size, light weight, immunity to electromagnetic interference (EMI) and corrosion, and embedding capability [8–12], and therefore they have been employed in monitoring of engineering structures worldwide. This paper will provide a comprehensive review on structural monitoring of civil infrastructure by use of the optical fiber sensing technology.

In the last two decades, a considerable number of investigations have been conducted in reviewing the progress of research and development of the optical fiber sensing technology as well as the applications of optical fiber sensors in the monitoring of various kinds of engineering structures [13–17]. Bhatia et al. [18] reported the progress in the performance and reliability of the optical fiber-based extrinsic Fabry-Perot interferometric (EFPI) strain sensor. Rao [19] gave a systematic review of progress on applications of FBG sensors in large composite and concrete structures, the electrical power industry, medicine, and chemical sensing. Leung [20] reviewed the applications of optical fiber sensors for monitoring of concrete structures. Measures et al. [21] overviewed the research on the development of structurally integrated optical fiber sensors for the smart structures. Merzbacher et al. [22] reviewed the strain monitoring of concrete structures by use of optical fiber sensors. López-Higuera et al. [23] summarized the main types of optical fiber techniques suitable for structural monitoring and introduced various optical fiber sensor-based engineering application scenarios. Ansari [24] provided a summary of basic principles pertaining to monitoring of civil engineering structures with optical fiber sensors. Majumder et al. [25] reviewed the recent research and development activities in structural monitoring using FBG sensors.

## 2. Fundamentals of Optical Fiber Sensors

Generally, an optical fiber sensor system consists of a light transmitter, a receiver, an optical fiber, a modulator element, and a signal processing unit. As the core part of an optical fiber sensor, the optical fiber is usually made from silica glass or polymer material, which itself can act as a sensing element or carry the light from the source to the modulator element. When the strain or temperature variation of the structure occurs, the surface-mounted or embedded optical fiber sensor in the structure will expand or contract. In accordance with the change of the length of the optical fiber, the optical fiber sensor modulates the light and reflects back an optical signal to the analytical unit for deriving the concerned physical quantity of the structure [26]. Based on the sensing principle, the optical fiber sensors can be categorized into different types as illustrated in the following sections [27].

### 2.1. Fiber Bragg Grating (FBG) Sensors

**Figure 1:**
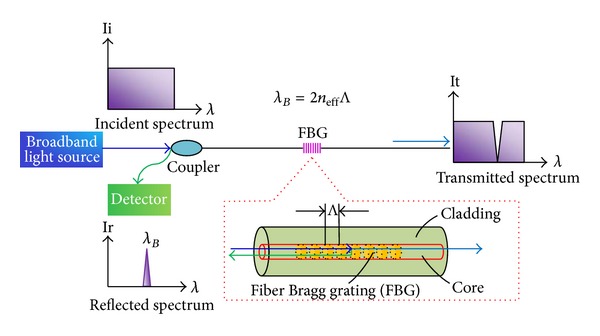
Measurement principal of FBG sensor.

Up to now, the FBG sensor has been widely used in the monitoring of civil engineering structures [28–32]. It can be regarded as a type of optical fiber sensor with varied refractive indices in the core. According to Bragg's law, a beam of white light is written in the FBG sensor, and when the light from the broadband source passes through the grating at a particular wavelength, the Bragg wavelength is reflected which is related to the grating period, as illustrated in Figure 1. The Bragg wavelength *λ*_*B*_ can be expressed by

(1) $$\begin{array}{c} \lambda_{B}=2n_{\text{eff}}\Lambda , \end{array}$$

where *n*_eff_ is the effective index of refraction and Λ is the grating period. The wavelength shift changes linearly with both strain and temperature. When the grating part is subjected to external disturbance, the period of the grating will be changed and the Bragg wavelength is varied accordingly. The variation of the Bragg wavelength can be obtained by

(2) $$\begin{array}{c} \Delta \lambda_{B}=\lambda_{B}\left\{\left(\alpha +\xi \right)\Delta T+\left(1-p_{e}\right)\Delta \varepsilon \right\}, \end{array}$$

where Δ*ε* is the strain variation, Δ*T* is the temperature change, *α* is the coefficient of the thermal expansion, *ξ* is the thermooptic coefficient, and *p*_*e*_ is the strain-optic coefficient.

### 2.2. Extrinsic Fabry-Perot Interferometric (EFPI) Sensors

**Figure 2:**
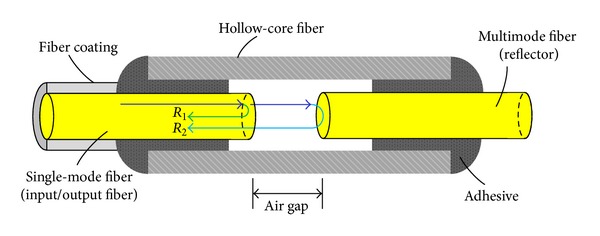
Measurement principal of EFPI sensor.

For an EFPI sensor, the optical fiber acts as the input or output path; the light from the source passes through the optical fiber to the sensing part and then to the demodulation system [33–38]. A typical EFPI sensor consists of the input/output fibers and the reflective fibers as well as a hollow-core tube for creating an air cavity, namely, the Fabry-Perot cavity. An adhesive is employed to bond the two components. As illustrated in Figure 2, the Fabry-Perot cavity is formed between an input single-mode fiber and a reflecting single-mode or multimode fiber, and two fibers are aligned inside a hollow-core tube. At both ends of the cavity, there are reflections on the uncoated ends of the fibers. *R*_1_ is referred to as the reference reflection which depends on the applied perturbation such as strain and temperature. *R*_2_ is the sensing reflection and depends on the length of the cavity, *L*. A sinusoidal output signal will be generated when *R*_1_ interferes with *R*_2_. Because the length of the cavity can be modulated by the applied perturbation, the EFPI sensor can be used to measure the applied perturbation according to the output signal. For the strain measurement, it can be expressed by

(3) $$\begin{array}{c} \varepsilon =\frac{\Delta l\left(\text{air}\;\mathrm{gap}\right)}{L}, \end{array}$$

where Δ*l* is the variation in the cavity.

### 2.3. Optical Time-Domain Reflectometry (OTDR) Sensors

**Figure 3:**
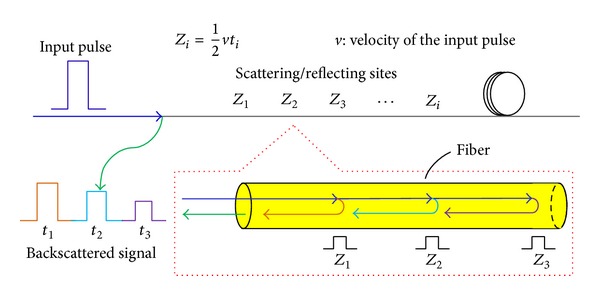
Measurement principal of OTDR sensor.

An optical time-domain reflectometry- (OTDR-) based sensor is capable of distributedly sensing along the length of an optical fiber with a specific refractive index [39, 40]. When a light pulse at a particular wavelength propagates along the optical fiber, the sensor can locate the position of the interaction according to the propagation time, as illustrated in Figure 3. The location of the variation of the measurand may be determined by the OTDR sensor. The OTDR-based distributed sensor is possible to be used to measure the change in the properties of the light along the entire optical fiber by measuring the time of flight of the returned pulses. The Brillouin optical time domain reflectometer (BOTDR) sensor is one of the distributed optical fiber sensors and is based on the Brillouin scattering. Due to the advantage of being capable of measuring continuous strain and temperature over a long distance, the BOTDR sensor has been widely applied in distributed monitoring of large-scale civil structures.

## 3. Innovation in Methodologies and Sensors

### 3.1. Improvement of Methods

Li et al. [41] proposed a theoretical model for describing the strain transfer relationship between the fiber core of the FBG sensor and the host material. Yun et al. [42] developed a new method based on the simulated annealing evolutionary algorithm for demodulation of the strain profile along an FBG distributed strain sensor. Imai and Feng [43] proposed a stress-transferring model incorporating drastic softening behavior for the surrounding components to investigate the stress transfer from a host structure to a sensing fiber. Zhang et al. [44] proposed a model reconstruction soft computing recognition algorithm based on genetic algorithm, support vector regression to achieve the reliability of the FBG-based sensor network. Gill et al. [45] presented a genetic algorithm for the inversion of Bragg grating sensor spectral data to determine the strain distribution along the grating. Prabhugoud and Peters [46] developed an integrated formulation for the calculation of the spectral response of an FBG sensor embedded in a host material system as a function of the loading applied to the host structure.

Liu et al. [47] proposed an adaptive filtering algorithm for the noise reduction and the detectability of seismic signals measured by an FBG measuring system. Ma et al. [48] presented a fast interrogation method for the dynamic and/or static strain gauge by use of a reflection spectrum from two superimposed FBGs. Jang et al. [49] developed a real-time impact localization algorithm for various composite structures using the impact-induced acoustic signals acquired by multiplexed FBG sensors. Schizas et al. [50] proposed a method for nonhomogeneous strain monitoring of composite structures with embedded wavelength multiplexed FBG sensors. Feng et al. [51] proposed a stationary wavelet transform method for signal processing of the distributed strain data from the BOTDR-based optical fiber sensors. Peters et al. [52] investigated the method of measurement of nonuniform strain field near the stress concentration by use of the embedded FBG sensor.

### 3.2. Development of Sensors

Lee et al. [53] developed an optical fiber accelerometer composed of a reflective grating panel and two optical fibers as transceivers which was capable of monitoring the low-frequency acceleration of large-scale civil engineering structures. Wang and Huang [54] developed an optical fiber corrosion sensor based on the principle of light reflection consisting of an optical fiber reflection sensor and a tube/film subassembly formed by welding a sacrificial metallic film to a steel tube. Rodriguez-Cobo et al. [55] designed an FBG-based smart structure embedded into composite laminates for simultaneous measurement of temperature and strain and conducted experimental investigations for performance validation. Pirozzi [56] developed a multipoint force sensor based on crossed optical fibers. Kim et al. [57] developed a gold-deposited extrinsic Fabry-Perot interferometer for dynamic strain measurement.

Cumunel et al. [58] investigated the capacity of continuously attached long-gauge optical fiber sensors for dynamic evaluation of structures. Gangopadhyay et al. [59] addressed different design and experimental packaging procedures of indigenously developed FBG sensors for strain measurement. Yuan et al. [60] presented an optical fiber two-dimensional sensing system for measuring the strain inside the concrete structure based on white-light Michelson interferometric optical fiber sensing technique. Liu et al. [61] designed a long-period fiber grating sensor for detecting the state of rebar corrosion in concrete. Yashiro et al. [62] proposed an embedded chirped FBG sensor for damage detection in the holed carbon fiber reinforced polymer (CFRP) laminate. Zhou et al. [63] designed an extrinsic Fabry-Perot interferometric strain sensor for damage evaluation of smart composite beams. Triollet et al. [64] proposed a superimposed FBG device to measure, localize, and discriminate strain and temperature effects simultaneously for structural monitoring.

Hong et al. [65] developed a distributed long-gauge FBG macrostrain sensor for condition assessment of reinforced concrete beams [66]. Quirion and Ballivy [67] validated the robustness of the Fabry-Perot optical fiber sensor in strain monitoring of the concrete structure. Davis et al. [68] developed an integrated FBG-based sensing system for broad area damage detection of composite structures. Villalba and Casas [69] evaluated the usefulness and effectiveness of the optical backscatter reflectometer sensor in damage detection of concrete structures. Torres et al. [70] presented a new FBG strain sensor with an unsymmetrical packaging configuration designed to be fixed to the surface of the monitored structure. Schröder et al. [71] developed a low-cost optical fiber sensing system for continuous on-site monitoring of contact forces in current collectors. Kim [72] developed a smart monitoring system for stress monitoring and damage detection of offshore structures using piezoceramic and optical fiber sensors.

Xu et al. [73] developed a novel Fabry-Perot optical fiber pressure sensor for application in high temperature environments. García et al. [74] developed a novel distributed optical fiber strain sensor suitable for long-distance condition monitoring of engineering structures. Liu et al. [75] developed a partially multiplexed EFPI-based optical fiber strain sensor system for in situ strain measurement of composite structures. Sun et al. [76] experimentally investigated the feasibility of corrosion monitoring of reinforced concrete structures by use of BOTDR sensors. Lan et al. [77] developed a combined Brillouin and FBG sensor for the monitoring of structural prestress loss in reinforced concrete beams. Zhou et al. [78] developed a smart fiber reinforced polymer (FRP) rebar with an embedded novel optical fiber for evaluation of prestress loss distribution in posttensioned concrete structures.

## 4. Applications of Optical Fiber Sensing Technology

### 4.1. Bridges

As the vital hinges of the transportation lines, the health conditions of the bridges have always been concerned by the bridge owners and managers. Continuous real*-*time monitoring of the environmental and operational loadings as well as the structural responses and behaviors of the bridges has been proved to be a promising and effective way for system identification, damage detection, safety condition assessment, and structural performance prediction. Due to the nonsubstitutable capabilities and unique advantages, the optical fiber sensing technology has been served as an effective tool for the monitoring of each phase of the bridge life-cycle (construction, operation, reinforcement, and rehabilitation), of various structural components of bridges (decks, towers, stay cables, suspenders, girders, piers, piles, and abutments), and of different measurands (strains, temperatures, accelerations, deflections/displacements, cracks, and corrosion). There have been a lot of investigations on bridge health monitoring and structural condition assessment based on the optical fiber sensing technology as detailed in the following sections.

#### 4.1.1. Integrated Bridge Monitoring System

A considerable number of optical fiber-based integrated SHM systems deployed on various types of bridges have been developed worldwide [79]. In USA, Mehrani et al. [80] developed a remote monitoring system based on optical fiber sensors for condition assessment of bridges and the performance of the developed system was validated through field instrumentation on a bridge in Florida, USA, during its construction stage. Glisic and Inaudi [81] developed a method for integrity monitoring of fracture critical bridges using simulated Brillouin scattering based on a crack or local deformation identification algorithm and a sensor delamination mechanism. Talebinejad et al. [82] developed an FBG-based accelerometer by use of the stiffness of the optical fiber and a lumped mass and the performance of which was evaluated during ambient vibration tests of a real bridge. In Canada, a total of 16 bridges have been instrumented with long-term SHM systems by intelligent sensing for innovative structures (ISIS) with various combinations of optical fiber sensors [83].

Brönnimann et al. [84] investigated the reliability and long-term stability of an FBG-based sensing and surveillance system through a monitoring period of six months during construction of a stay cable in Switzerland. In Portugal, Rodrigues et al. [85] developed an FBG-based system with embedded displacement and strain transducers for long-term monitoring of structural performance of concrete bridges which was applied to a concrete bridge. Barbosa et al. [86] developed a novel weldable FBG sensing system for strain and temperature monitoring of steel bridges and for loading tests and health monitoring of a circular pedestrian steel bridge. In UK, Kerrouche et al. [87] developed a relatively cheap and effective sensing system using a compact FBG-based monitoring system incorporating a scanning Fabry-Perot filter, and the performance of the system was validated through laboratory experiments and field tests in a real bridge. Kister et al. [88, 89] conducted the research on structural monitoring of a composite road bridge by use of FBG sensors, and the performance of the adhesives and the protection system of the sensors were evaluated through field pullout tests. Mokhtar et al. [90] created an innovative FBG-based sensor system for accurate strain measurement with full temperature compensation towards condition monitoring and assessment of arch bridges. Surre et al. [91] developed an optical fiber sensor system for long-term strain monitoring and condition assessment of a redundant 50-year-old concrete footbridge.

In Hong Kong, the high-speed demultiplexing/interrogation system for FBG sensor arrays and FBG sensor package units were deployed for long-term monitoring on the Tsing Ma Bridge which is the world's longest suspension bridge carrying both highway and railway traffic [92]. Yau et al. [93] proposed a simple, inexpensive, and practical method for measurement of the vertical displacement of bridges by use of FBG sensors. In Chinese mainland, Zhao et al. [94] integrated the distributed Brillouin optical time domain analysis (BOTDA) technology and the FBG sensing technology for strain monitoring of bridges. In Korea, Chung et al. [95] conducted the experimental study on the applicability of long-gauge optical fiber sensors for the monitoring of the structural defection of the prestressed concrete bridges. Lin et al. [96] developed an FBG-based sensing system for online monitoring of highway bridges during construction to record the hydration effects, curing periods, prestressing responses, and removal of support frames.

#### 4.1.2. Monitoring of Rehabilitated and Antique Bridges

Research efforts also have been devoted to measuring the structural behaviors of old bridges or deficient bridges during rehabilitation by use of the optical fiber sensing system [97]. Jiang et al. [98] applied two types of optical fiber sensors embedded in FRP material to monitor the global and local behaviors of the strengthened bridge structures. Zhang et al. [99] introduced two types of optical fiber sensing technologies (FBG and BOTDR) for health monitoring of rehabilitated reinforced concrete girder bridges, and the static and dynamic loading tests were carried out with a simply supported reinforced concrete T-beam strengthened by externally posttensioned aramid fiber reinforced polymer (AFRP). Costa and Figueiras [100] presented the design of an advanced FBG-based monitoring system which was applied to a century steel arch bridge in Portugal.

#### 4.1.3. Monitoring of Bridge Cables and Suspenders

He et al. [101] carried out an investigation on cable force monitoring by use of the local high-precision FBG sensor in combination with the distributed BOTDA sensing technique. Li et al. [102] developed a smart stay cable assembled with FBG-based strain and temperature sensors which were incorporated into a glass fiber reinforced polymer (GFRP) bar. The efficiency of the developed smart stay cable was proved by application to evaluate the fatigue accumulative damage of a stay cable bridge in China [103, 104].

#### 4.1.4. Bridge Scour Monitoring

Zhou et al. [105] proposed an FBG sensing system for scour monitoring of foundations of bridge piers and abutments. This developed system introduced a uniform-strength FRP beam instrumented with two FBG sensors in two sides of the neutral axis, and the feasibility of the system was validated through laboratory tests. Lin et al. [106, 107] developed two types of FBG-based systems for real-time bridge scour monitoring, which were capable of measuring the process of scouring/deposition and the variation of the water level. The in situ FBG scour monitoring system was demonstrated to be robust and reliable for real-time scour-depth measurement and to be valid for indicating the depositional depth. Xiong et al. [108] developed a bridge scour monitoring system by use of FBG sensors, and the experimental investigations verified that the recommended scour monitoring system was capable of measuring the water level, the scour depth, the entire process of scour development, and the deposition height due to the refilling process.

### 4.2. Buildings

The optical fiber sensing technology has been employed in safety condition monitoring of the high-rise structures during in-construction and in-service stages. Bastianini et al. [109] utilized the embedded optical fiber Brillouin sensors for strain monitoring and crack detection of a historical building. Antunes et al. [110] conducted dynamic monitoring of a reinforced concrete water reservoir and a slender metallic telecommunication tower by use of FBG-based biaxial accelerometers. Ni et al. [111] deployed massive FBG sensors for strain and temperature monitoring of the Canton Tower. Li et al. [112] performed an investigation on the feasibility of the FBG-based monitoring system instrumented in an 18-floor tall building during construction. The FBG sensors were used to monitor the strain and temperature of the building in three steps of construction, that is, before the concrete pouring, during the pouring and curing of concrete, and the construction of subsequent upper floors of the building.

### 4.3. Tunnels and Pipelines

Ye et al. [113] addressed two engineering paradigms on safety monitoring of tunnel construction by use of FBG sensors. Metje et al. [114] presented a new optical fiber sensing system for structural displacement monitoring which was successfully applied to measure the displacement of a tunnel lining. The novel system was based on a square fiberglass smart rod which was proved sensitive enough to measure the rotational movement of 0.5° and the lateral movement of 0.1 mm of the fixings. Li et al. [115] developed a metal groove encapsulating technique for the bare FBG sensor to measure the surface strain of the second lining of the tunnel. Li et al. [116] developed a differential FBG strain sensor for monitoring the stability of the tunnel during the backfilling and traffic-operating periods.

Glisic and Yao [117] proposed a method for real-time, automatic, or on-demand assessment of health conditions of buried pipelines after the earthquake based on distributed optical fiber sensors, the research of determination of sensor topologies, selection and development of sensors, development of installation and implementation procedures, and large-scale tests were conducted. Zhang et al. [118] experimentally investigated the prediction of locations and progression sequences of the pipe buckling with the aid of the broadening factor of the Brillouin spectrum width using high strength carbon-coated fibers and standard communication fibers.

### 4.4. Wind Turbines

A review of the current status and a discussion on research and implementation of FBGs and long-period gratings in wind turbine blade sensors can be found in [119]. Arsenault et al. [120] developed an FBG-based distributed strain sensor system for real-time monitoring of a wind turbine and conducted the validation tests under a laboratory scale under various loading conditions. Kim et al. [121] conducted experimental investigations on deflection estimation of wind turbine blades using embedded FBG sensors. Burgmeier et al. [122] developed and tested an FBG-based sensor system for remote measurement of strain that affects the power cable in an offshore wind turbine.

Bang et al. [123] introduced an FBG-based arrayed sensor system for measurement of strain and bending deflection of wind turbine towers. Ge et al. [124] developed a specific intensity-modulated optical fiber accelerometer for vibration monitoring of wind turbine blades. Schroeder et al. [125] installed an FBG measurement system for load monitoring in horizontal-axis wind turbines. Choi et al. [126] determined the tip deflections of a composite wind turbine blade through a static load test using mechanical strains measured by FBG sensor probes.

### 4.5. Railway Infrastructure

Recently, the optical fiber sensor-based monitoring system has attracted great interests among the researchers in the fields of railway engineering and optical engineering. Yan et al. [127] proposed three FBG-based methods for strain measurement and axle counting in high-speed railway systems, and the advantages and limitations of these approaches were discussed in terms of feasibility and cost-effectiveness through laboratory verification and evaluation. Wei et al. [128, 129] described a real-time wheel defect detection system through deploying FBG sensors on rail tracks of the Hong Kong mass transit railway (MTR) to gain the track strains upon wheel-rail interaction and generate a reliable condition index reflecting the wheel condition, and the effectiveness of the introduced system was verified by extensive field tests. Filograno et al. [130] implemented an FBG-based railway security monitoring system on the Spanish high-speed line Madrid-Barcelona for train identification, axle counting, speed and acceleration detection, wheel imperfection monitoring, and dynamic load calculation.

Pimentel et al. [131] developed a hybrid fiber-optic/electrical train characterization system with a new weight-in-motion (WIM) algorithm for on-motion determination of the train speed, acceleration and weight distribution for traffic monitoring, and safety evaluation of a railway bridge in Portugal. Kerrouche et al. [132, 133] conducted the research on structural monitoring of a decommissioned concrete railway bridge in Sweden loaded to failure by use of an FBG-based distributed sensor system.

Kang and Chung [134] developed an integrated FBG-based monitoring scheme for a maglev guideway in Korea to measure the parameters involving strains, curvatures, vertical defections, and frequencies which were compared with those obtained from the conventional sensors [135]. Yoon et al. [136] proposed a distributed Brillouin optical correlation domain analysis- (BOCDA-) based sensing system to measure the longitudinal strain distribution of a rail in real time, and the results of a spatial resolution of 3.8 cm and an accuracy of ±15 *με* were achieved under different loading conditions applied to a 2.8 m rail. Wang et al. [137] utilized* A*-thermal FBG sensors and electronic sensors to record performance changes in the continuous welded rail, and the monitoring results revealed that the optical fiber sensor was durable and capable of long-term monitoring and was capable of providing sensitive, clear, and stable signals.

Bocciolone et al. [138] presented the application of FBG sensors on a pantograph for monitoring of the contact force and the vertical acceleration of the pantograph head of the pantograph-catenary system in an underground line. Boffi et al. [139] developed an innovative optical fiber sensor-based system for online monitoring of the contact force between the pantograph and the catenary at low and high frequencies.

### 4.6. Geotechnical Structures

Regarding the applications in geotechnical engineering, Pei et al. [140, 141] developed an FBG-based in-place inclinometer for lateral displacement measurement of slopes in accordance with the classical indeterminate beam theory which was successfully installed in a slope in China for long-term displacement monitoring. Kister et al. [142] deployed FBG sensors in reinforced concrete foundation piles for strain and temperature monitoring and structural health condition assessment. Lu et al. [143] conducted the field measurement of the stress within the precast pile by use of the BOTDR-based optical fiber sensing technique. Kim et al. [144] developed a specially designed FBG-embedded tendon for the monitoring of the prestress force and load transfer of the ground anchor and the feasibility of the device was verified through laboratory and field tests. Legge et al. [145] developed an FBG-based stress cell for determination of the full state of three-dimensional stress at any accessible or predetermined point in a soil mass or structure.

## 5. Conclusions

This paper provides a summary of the research and development in the area of structural monitoring of civil infrastructure by use of the optical fiber sensing technology. Based on a comprehensive review of the optical fiber sensor-based theories, methods, technologies, and applications, the following concluding remarks are made: (i) due to their unique merits, the optical fiber sensors have been widely used in life-cycle monitoring of civil infrastructure such as bridges, buildings, tunnels, pipelines, wind turbines, railway infrastructure, and geotechnical structures; (ii) the optical fiber sensing technology is capable of measuring lots of types of measurands such as strains, temperatures, accelerations, deflections/displacements, cracks, and corrosion; and (iii) the exploitation of protection measures in sensor installation as well as the development of cost-effective optical fiber demodulation instruments are desirable in the further research.
